# Cost-effectiveness of radiofrequency ablation *versus* percutaneous ethanol injection for early hepatocellular carcinoma in a resource-poor setting: a randomized trial

**DOI:** 10.31744/einstein_journal/2024GS0683

**Published:** 2024-09-25

**Authors:** Guilherme Cayres Mariotti, Guilherme Eduardo Gonçalves Felga, Rodrigo Gobbo Garcia, Priscila Mina Falsarella, Bruno Pagnin Schmid, Daniel Tavares Malheiros, Ronaldo Hueb Baroni, Ary Serpa

**Affiliations:** 1 Hospital Israelita Albert Einstein Department of Interventional Radiology São Paulo SP Brazil Department of Interventional Radiology, Hospital Israelita Albert Einstein, São Paulo, SP, Brazil.; 2 Hospital Israelita Albert Einstein Liver Transplant Department São Paulo SP Brazil Liver Transplant Department, Hospital Israelita Albert Einstein, São Paulo, SP, Brazil.; 3 Hospital Israelita Albert Einstein Hospital Municipal da Vila Santa Catarina Dr. Gilson de Cássia Marques de Carvalho São Paulo SP Brazil Hospital Municipal da Vila Santa Catarina Dr. Gilson de Cássia Marques de Carvalho; Hospital Israelita Albert Einstein, São Paulo, SP, Brazil.; 4 Hospital Israelita Albert Einstein Value Management Office São Paulo SP Brazil Value Management Office, Hospital Israelita Albert Einstein, São Paulo, SP, Brazil.; 5 Hospital Israelita Albert Einstein Radiology Department São Paulo SP Brazil Radiology Department, Hospital Israelita Albert Einstein, São Paulo, SP, Brazil.; 6 Monash University School of Public Health and Preventive Medicine Australian and New Zealand Intensive Care Research Centre Melbourne Australia Australian and New Zealand Intensive Care Research Centre (ANZIC-RC), School of Public Health and Preventive Medicine, Monash University, Melbourne, Australia.; 7 University of Melbourne Melbourne Medical School Department of Critical Care Melbourne Australia Department of Critical Care, Melbourne Medical School, University of Melbourne, Austin Hospital, Melbourne, Australia.

**Keywords:** Liver neoplasms, Radiology, interventional, Ablation techniques, Liver transplantation, Cost-effectiveness evaluation

## Abstract

**Objective:**

This study assessed the cost-effectiveness of radiofrequency ablation compared with percutaneous ethanol injection in patients with early hepatocellular carcinoma in relation to the objective response rate and costs related to the procedure.

**Methods:**

This was a prospective single-center randomized trial. The primary outcome was cost-effectiveness. Secondary outcomes were the complete response rate according to the modified response evaluation criteria in solid tumors 60 days after randomization and the complication rate within 180 60 days.

**Results:**

Fifty patients were placed into the following groups: percutaneous ethanol injection (n=23) and radiofrequency ablation (n=27). Fifty-four nodules were randomized (mean follow-up: 205.37 days). The estimated mean hospital cost was US$ 1854.11 and US$ 2770.96 for the Radiofrequency Ablation and Percutaneous Ethanol Injection Groups, respectively. The incremental cost-effectiveness ratio was US$ -2674.59, which is advantageous for radiofrequency ablation. After 60 d, 28 of 29 nodules in the Radiofrequency Ablation Group achieved complete response *versus* 12 of 22 in the Percutaneous Ethanol Injection Group (RD, 42.01 [95%CI= 20.55–63.24]; p<0.001). Only four early complications were observed among patients treated by percutaneous ethanol injection (p<0.05). Late complications occurred in two and one patient(s) in the Radiofrequency Ablation and Percutaneous Ethanol Injection Groups (p>0.05), respectively.

**Conclusion:**

Radiofrequency ablation was more cost-effective and achieved higher complete response and lower complication rates than the Percutaneous Ethanol Injection Group within this cohort.

**Registry of Clinical Trials:**

NCT06450613

## In Brief

Mariotti et al. demonstrated that radiofrequency ablation is more cost-effective, achieves a higher complete response rate, and has a lower complication rate than percutaneous ethanol injection in patients with early-stage hepatocellular carcinoma.

## Highlights

◼ Radiofrequency ablation and percutaneous ethanol injection are neoadjuvant hepatocellular carcinoma treatment methods.
◼ Radiofrequency ablation achieves a higher complete response rate (96.3% versus 60.0%, p=0.001).
◼ Total cost analyses showed radiofrequency ablation as a more expensive method (p<0.001).
◼ The incremental cost-effectiveness ratio was US$ -2674.59 with an advantage for radiofrequency ablation.
◼ Incremental effectiveness (34.2%) was in favor of radiofrequency ablation versus percutaneous ethanol injection.

## Graphical abstract

**Figure:**
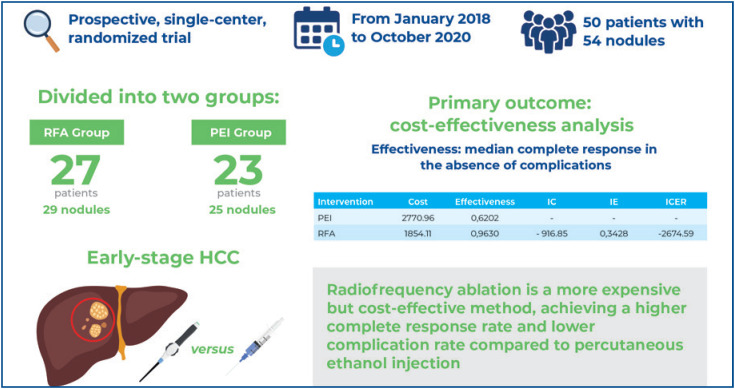

## INTRODUCTION

Local ablative procedures play a key role in the management of hepatocellular carcinoma (HCC) by enabling downsizing, improving prognosis after liver transplantation (LT), and reducing dropouts from the waiting list.^(1-3)^ Radiofrequency ablation (RFA) and percutaneous ethanol injection (PEI) are two nonsurgical neoadjuvant alternatives.^(4)^ Thermal ablation with radiofrequency is the standard of care for patients with BCLC 0 and A tumors that are not suitable for surgery. However, PEI is an option in cases where thermal ablation is not technically feasible, especially for tumors measuring <2cm.^(5)^

In recent studies, RFA has demonstrated a higher 3-year overall survival rate and enhanced local disease control in patients with HCC and nodules up to 3cm in size than those treated with PEI.^(4,6)^ However, the most cost-effective method remains controversial, especially in resource-poor settings. Additionally, cost analysis can mitigate unfounded perceptions of "higher costs" driving therapeutic allocation, especially in environments where resource allocation is crucial.

## OBJECTIVE

To assess the cost-effectiveness of radiofrequency ablation *versus* percutaneous ethanol injection in adult patients with early hepatocellular carcinoma.

## METHODS

### Trial design

This was a pilot, single-center, randomized, open-label trial with two parallel arms. This study was conducted in accordance with the principles of the Declaration of Helsinki. Written informed consent was obtained from all patients included in the study, and the study protocol was approved by the institutional review board (CAAE: 68277917.4.0000.0071; 3903459). The Consolidated Health Economic Evaluation Reporting Standards (CHEERS) was used to evaluate the health economics.^(7)^

### Eligibility criteria for patient selection

Patients admitted to a tertiary public hospital in Brazil (*Hospital Municipal da Vila Santa Catarina Dr. Gilson de Cássia Marques de Carvalho*) with early HCC within the Milan criteria, listed for LT, and with indications for neoadjuvant treatment were eligible for enrollment. The following inclusion criteria were considered: 1) single or multiple HCCs measuring 2–3cm in accordance with the American Association for the Study of Liver Diseases (AASLD), 2) Child–Pugh grade A or B, 3) Barcelona Clinic Liver Cancer Staging A (BCLC-A), 4) fulfillment of the national criteria for LT, and 5) indication for neoadjuvant treatment.

Exclusion criteria were: 1) vascular invasion or extra-hepatic dissemination; 2) refractory or intractable ascites; 3) hepatic encephalopathy grade 3 or 4; 4) complete portal vein thrombosis; 5) bilirubin level >3mg/dL; 6) coagulation disorders (defined as platelet count < 20×10^9^/L and/or international normalized ratio >2); 7) moderate to severe hydrothorax; 8) creatinine clearance <30mL/min; 9) Model for End-stage Liver Disease score >30; 10) expected technical problems for either PEI or RFA; 11) patient's refusal to sign informed consent.

### Randomization and masking

The patients were randomized in a 1:1 ratio to undergo ablation using PEI (PEI Group) or RFA (RFA Group). Local investigators performed randomization using a central, dedicated, password-protected, encrypted, web-based automated randomization system, and the allocation list was prepared by an independent statistician. Randomization was conducted individually to reduce variability and potential confounding factors between the two groups.

### Interventions

Radiofrequency ablation and PEI were performed in the angiography suite under ultrasound (US) guidance using 16-slice multidetector computed tomography (CT). All procedures were performed percutaneously by two experienced interventional radiologists (10–20 years of experience). Anesthesia modalities included general anesthesia with intravenous sedation and local anesthesia.

Radiofrequency ablation was performed in a single session using an RFA 200-W generator device. The electrode had a 17-gauge single needle with 3.0cm exposed active parts or a 17-gauge triple-cluster needle with 2.5cm exposed active parts, both 15 and 20cm in length, with internally cooled applicators.

A radiofrequency current was emitted for 12 minutes in each cycle, with CT-scans and US performed after each cycle to assess the ablation margins (>1cm). If the scan showed incomplete ablation, multiple overlapping RFA ablations were performed to achieve complete tumor ablation. Preprocedural hydrodissection with a 5% glucose solution using an 18-gauge Chiba needle was performed if any unwanted structure was adjacent (<2cm) to the tumor. Contrast-enhanced CT scans (using 1.7ml/Kg of Iohexol GE Healthcare^®^; Shanghai, China) were performed at the end of each procedure to assess complications and ablation margins. Track ablation was routinely performed (Figure 1S, Supplementary Material).

Percutaneous ethanol injection was performed in multiple sessions (up to three, with a 1-week interval) using a 15- or 20cm long, 22-gauge Turner needle. An injection of absolute ethanol was administered following a contrast-enhanced CT scan using a 20% iodinated contrast solution to evaluate adequate positioning of the needle. The injected volume was based on the tumor volume estimated from the preprocedural CT scan, assuming a 1:1 ratio (range: 4–20mL). The needle was repositioned several times to obtain homogeneous perfusion inside the nodule (homogeneous hyperechogenic appearance on US at the end of treatment; Figure 2S, Supplementary Material).

Patients who underwent RFA were routinely admitted to the hospital following the procedure, aiming for hospital discharge 1 day after the procedure. Patients who underwent PEI were discharged from the hospital 6 hours after the procedure if no complications were noted.

The follow-up protocol included regular clinical assessments and magnetic resonance imaging (MRI) 60, 120, and 180 days after the procedure. All MRI scans were reviewed by a team of unblinded radiologists who were unrelated to the study.

### Outcomes

The primary outcome was cost-effectiveness. The effectiveness parameter was the complete response rate in the absence of complications, and the cost of hospitalization was determined by reviewing the billing details of hospitalization expenses. Costs are the exact costs from all hospital stays (hospital stay costs, supply costs, lab costs, nursing staff, anesthesia, and diagnostic imaging exams), which were calculated and reported in U.S. dollars (US$). The year of conversion was 2021. Transportation costs were not included.

The study was conducted in a public health system managed by a private practice hospital with a nonprofit organization contract. Therefore, no bidding was performed, and the price tag was defined in the same way as transactions between medical corporations and private hospitals. There were no donations.

Effectiveness was expressed as the treatment success rate without complications. The incremental cost-effectiveness ratio (ICER) was calculated by dividing the difference in total cost (incremental cost) by the difference in incremental effectiveness. The ICER is expressed as the ratio of incremental costs to incrementally avoided complications. A sensitivity analysis was performed to measure the uncertainty in relation to the ICER. The lower and upper limits of each model variable (probability, cost, and effectiveness) were measured. The sensitivity analysis is presented in a tornado diagram (Figures 3S, Supplementary Material). As this was a real-world study, the lower limit was considered the lowest cost value, and the upper limit was considered the highest cost. All the effectiveness variables were simulated with a variation from 0 (no effectiveness) to 1 (maximum effectiveness). The probabilities were also simulated with 20% variation for the lower and upper limits.

Safety was assessed by analyzing early (<7 days) and late (≥7 days) complications.

Complications included portal vein thrombosis, bleeding, hepatic encephalopathy, infectious complications, neoplastic dissemination adjacent to the lesion, and late recurrence.

A cost-effectiveness analysis was performed using TreeAge Pro 2017 (TreeAge Software, Inc., Williamstown, MA, USA). The time horizon was 180 days, and the willingness to pay limit was 1 × Gross Domestic Product per capita.

Secondary outcomes were: 1) complete response rate according to the mRECIST criteria 60 days post-randomization (defined as the disappearance of any intratumoral arterial enhancement in all target lesions) based on independent radiologic review;^(8)^ 2) rate and grade of complications related to the methods; and 3) late recurrence between 120 and 180 days post-treatment.^(7)^

### Statistical analysis

It was estimated that 50 patients would be required to reduce imprecision regarding the incidence of the primary outcome.^(9)^ The power calculation estimated that the effect size difference was 98% (based on the complete response according to mRECIST). All the data were collected by a trained research assistant using an electronic database. Continuous variables were reported as median (quartile 25–75%) and compared using Wilcoxon rank-sum tests. Categorical variables were reported as numbers and percentages and compared using Fisher's exact test. Patients were analyzed according to their randomization group, and the analysis dataset included all patients who were randomized and underwent the entire procedure.

Costs were compared using Wilcoxon rank-sum tests and complications were compared using Fisher's exact tests. Binary secondary outcomes were compared between groups using a generalized linear model considering a binomial distribution and an identity link and reported as risk difference (RD) and 95% confidence interval (95%CI). Sensitivity analysis was performed to evaluate the effect of model assumptions and parameters on the results and is detailed in a tornado diagram (Figure 3S, Supplementary Material).

The significance level for the primary and secondary outcomes was 0.05, without adjustment for multiple comparisons. The primary outcome analysis was exploratory in nature. Reported p values were 2-sided, and only a complete case analysis was carried out. All analyses were performed using the R software, version 4.0.2 (R Core Team).

## RESULTS

Fifty-four patients with 58 nodules were randomized between January 2018 and October 2020. After exclusion, the data from 50 patients with 54 nodules were included in the primary analysis: 23 in the PEI and 27 in the RFA Groups (Figure 1). Baseline characteristics were well balanced between the groups (Table 1). The trial ended after the sample size was reached and a complete follow-up protocol was achieved. The mean follow-up time was 205.37 days (standard deviation, 61.72; range: 63–323).

**Figure 1:**
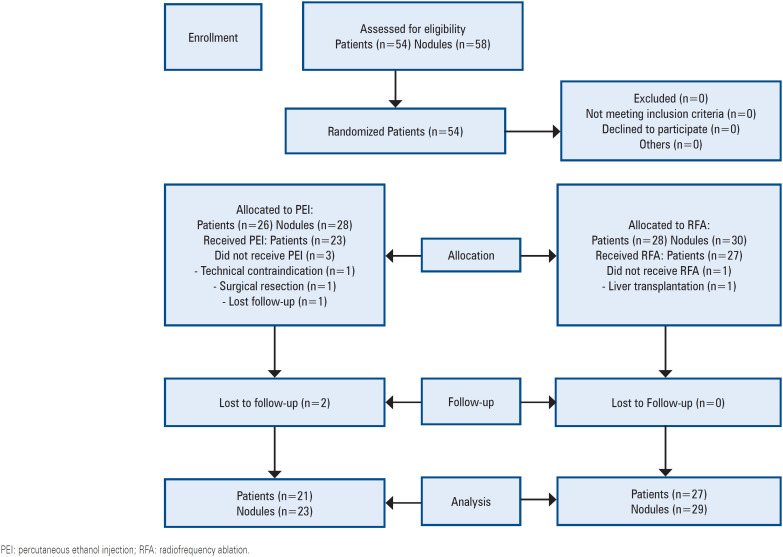
Flow diagram of study participants (CONSORT diagram)

**Table 1:** Demographic and clinical data for patients in the PEI and RFA Groups

		RFA Group (n=27)	PEI Group (n=23)
Age (years)		62 (55 – 68)	63 (57–68)
Male n (%)		24 (88.9)	17 (73.9)
Child-pugh grade A		20 (74.1)	15 (65.2)
Child-pugh grade B		7 (25.9)	8 (34.8)
Cirrhosis etiology			
	Hepatitis B viral infection	1 (3.7)	2 (8.7)
	Hepatitis C viral infection	13 (48.1)	11 (47.8)
	Alcohol	9 (33.3)	7 (30.4)
	Cryptogenic	4 (14.8)	3 (13.0)
Nodules characteristics			
	Number/group	29	25
	Number/patient	1.0 (1.0–1.0)	1.0 (1.0–1.0)
	Tumor size (cm)	2.1 (2.0–2.6)	2.2 (2.0–2.5)
	Serum bilirubin (mg/dL)	1.4 (0.9–2.0)	1.2 (0.7–2.3)
	Serum albumin (g/dL)	3.7 (3.2–4.3)	3.7 (3.4–3.9)
	Platelet count (× 10^9^/L)	84 (63–121)	99 (66–120)
	Serum AFP level (mg/mL)	5.8 (3.6–27.7)	8.0 (4.3–72.9)
	International normalized ratio	1.3 (1.2–1.5)	1.3 (1.2–1.4)

Data are reported as median (25%–75% quartile) or number (percentage).

AFP: alpha-fetoprotein; PEI: percutaneous ethanol injection; RFA: radiofrequency ablation.

### Primary outcome

The use of RFA was associated with an additional initial procedure cost, mainly due to the price of the RFA needle (p=0.003). In addition, the total cost analyses showed that RFA was the more expensive method (p<0.001; Table 2). However, in the cost-effectiveness analysis, the ICER was US$ -2674.59 which is advantageous for RFA. The efficacy (median complete response in the absence of complications) was 96.3% for RFA and 62.0% for PEI. The incremental effectiveness was 34.2% in favor of RFA *versus* PEI. A complete cost-effectiveness analysis is presented in table 3 and figures 2 and 3.

**Table 2:** Cost analysis in each group

	RFA Group (n=27)	PEI Group (n=23)	p value
Cost- procedure			
Median (quartile 25–75%)	768.20 (626.53–1349.02)	437.58 (215.73–749.12)	<0.003
Mean ± SD	1107.85±854.90	701.74±838.56	
Minimum/ Maximum	448.31 / 3659.75	51.31 / 3082.64	
Cost- hospitalization			
Median (quartile 25–75%)	365.36 (82.23–574.95)	0.0 (0.0–0.0)	<0.001
Mean ± SD	468.68±449.23	989.94±4747.58	
Minimum/ Maximum	0.0 / 1696.53	0.0/ 22768.61	
Cost- follow-up MRI			
Median (quartile 25–75%)	229.75 (167.37–262.91)	217.49 (111.15–267.08)	>0.465
Mean ± SD	221.82±94.99	195.79±111.77	
Minimum/ Maximum	55.22 / 511.75	0.0 / 454.47	
Cost- Total			
Median (quartile 25–75%)	1583.48 (1317.76–2092.36)	578.41 (403.96–1089.87)	<0001
Mean ± SD	1803.78±887.74	1896.48±4815.84	
Minimum/ Maximum	522.91 / 4294.88	150.51 / 23638.88	

Costs were expressed in U.S. dollars (US$) considering the average exchange rate for the month of inclusion of the last patient - Brazilian currency (BRL real [R$]; US$1 = R$ 5.63). p<0.05 is considered significant; p<0.01 is highly significant.

MRI: magnetic resonance imaging; PEI: percutaneous ethanol injection; RFA: radiofrequency ablation; SD: standard deviation.

**Table 3:** Analysis of cost-effectiveness in each group

Intervention	Cost	Effectiveness	IC	IE	ICER
PEI	2770.96	0,6202	–	–	–
RFA	1854.11	0,9630	- 916.85	0,3428	-2674.59

Costs are expressed in U.S. Dollars (US$).

IC: incremental cost; ICER: Incremental cost-effectiveness ratio; IE: Incremental effectiveness; PEI: percutaneous ethanol injection; RFA: radiofrequency ablation.

**Figure 2:**
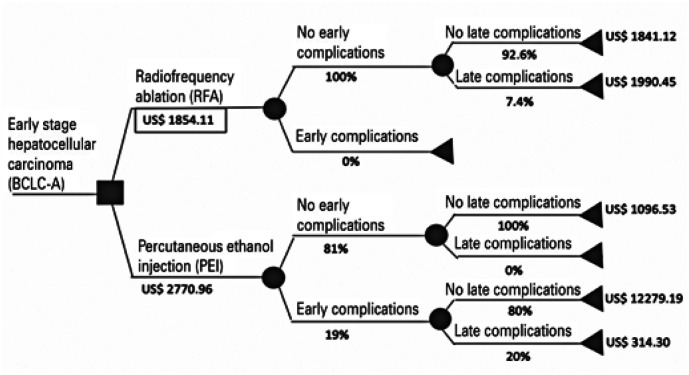
Probabilistic decision tree with mean hospital costs. Therapeutic strategies are shown after decision node (to the right of ■). Probabilistic outcome are shown after chance nodes (to the right of ●). Terminal nodes are represented by ◀). The values within the rectangles represent the dominant strategy (less costly and more effective)

**Figure 3:**
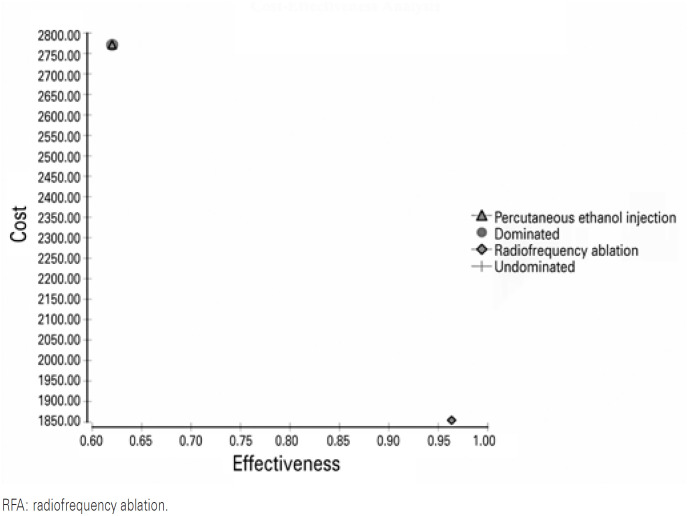
Cost-effectiveness analysis showing radiofrequency ablation as the dominant technique

### Secondary outcomes

After 60 d, 26 of 27 (96.3%) patients in the RFA Group achieved complete response *versus* 12 of 20 (60.0%) patients in the PEI Group (RD, 36.30 [95%CI= 14.50 to 58.85]; p=0.001). Comparing the nodules, 28 of 29 (96.5%) nodules in the RFA Group achieved complete response *versus* 12 of 22 (54.5%) in the PEI Group (RD, 42.01 [95%CI= 20.55 to 63.24]; p<0.001). The complete objective response rates for each group is presented in Table 1S, Supplementary Material. Eleven patients were bridged to the transplant group (RFA Group, n=7; PEI Group, n=4).

The incidence of complications was low in both groups.^(10)^ During short-term follow-up, patients with RFA presented with no complications, while four patients in the PEI Group presented with adverse events (AEs) (two cases of temporary segmental portal thrombosis, one case of transient hepatic encephalopathy, and one case of perihepatic abscess and pleural empyema). Late complications occurred in two patients in the RFA Group (one case of late recurrence and one case of possible neoplastic dissemination adjacent to the lesion, which may be related to hydrodissection posterior to the lesion or the growth of satellite nodules adjacent to the target lesion) and one patient in the PEI Group (late recurrence). Late complications did not prevent patients from continuing on the liver transplant list, as they remained within the Milan criteria with better conditions.^(10)^

Monte Carlo simulation (probabilistic sensitivity) analysis also showed a statistically significant difference between the two groups regarding the complete response rate according to mRECIST (p<0.001)^(7)^ (Table 2S and Figure 3S, Supplementary Material).

## DISCUSSION

In this pilot randomized clinical trial, RFA achieved superior cost-effectiveness compared to PEI for the treatment of early HCC. Although, initially, it is a more expensive procedure, RFA presents a higher complete response and lower complication rates and costs than PEI. The metrics observed in cost-effectiveness studies may allow more assertive choices for value-based medicine in the treatment of early HCC, resulting in better outcomes and greater sustainability of the healthcare system, especially in developing countries.

It is important to emphasize that possible complications, which may lead to prolonged hospitalization, may have a substantial effect on total costs, rather than solely by the procedure itself. This was observed in a case of PEI in which the total hospitalization cost (US$ 22768.61) was 20-fold greater than the mean hospitalization cost (US$ 989.94) for the cohort.

This outlier had a decisive impact on the final cost analyses, and although it could have occurred in the RFA Group, this thermal ablation method had the potential to be more cost-effective than PEI.

The comparison of RFA and PEI in relation to radiological response and survival rate in the treatment of early HCC has been debated, with conflicting results. A randomized clinical trial comparing both methods in 139 cirrhotic patients with Child-Pugh classes A/B and HCC showed that there was an incremental healthcare cost of US$ 9012.56 for each additional patient treated with RFA.^(11)^ Similar results were reported in another trial that analyzed 285 patients with single HCC, demonstrating that PEI was a much cheaper method.^(12)^ However, some authors have suggested that the overall cost-effectiveness of RFA requires further evaluation.^(6)^The present study demonstrated that despite being an initially more expensive procedure, RFA has lower complication rates and costs. Overall, these findings should be considered when selecting a cost-effective ablation method for the management of patients with early HCC.

Radiofrequency ablation has several advantages over PEI. First, it allows for complete treatment in a single section of selected patients.^(4)^ Second, the results of the present study showed a higher radiological response rate, which corroborates the findings of previous studies. However, PEI is still a valuable option as a neoadjuvant therapy for early HCC and should be considered when RFA is not available or in situations in which RFA presents some limitations, such as tumors near the bowel, where hydrodissection is unsatisfactory, and in close proximity to the biliary system or major vessels.^(13)^ In the present study, only one case of incomplete ablation due to the heat sink effect occurred, in which repeated RFA was attempted to achieve complete tumor ablation, highlighting the importance of considering these anatomical patterns during treatment planning.

This study had several limitations, including its single-center design and small sample size. The MRI scans were reviewed by an unblinded team. Treatment costs can vary among institutions over time, possibly leading to different results. Late recurrence was included as a late complication and information for which individual interpretations may vary. In addition, this study was not performed with HCC <2cm, where ethanol ablation seems to be more effective.

Additionally, long-term quality of life and overall survival rates were not assessed. Microwave ablation has become a feasible treatment for HCC in addition to RFA; however, it has not yet been analyzed.^(14)^ Finally, one patient who was administered PEI presented with a liver abscess and pleural empyema that was surgically managed, requiring a long hospital stay, thereby resulting in high costs and explaining the one outlier in the cost analysis.

## CONCLUSION

The cost-effectiveness analysis points to radiofrequency ablation as a possible strategy in patients with early hepatocellular carcinoma, considering the complete response rate in the absence of complications as a parameter of effectiveness and the probabilities of complications and hospital costs.

## Figures and Tables

**Figure 1S:**
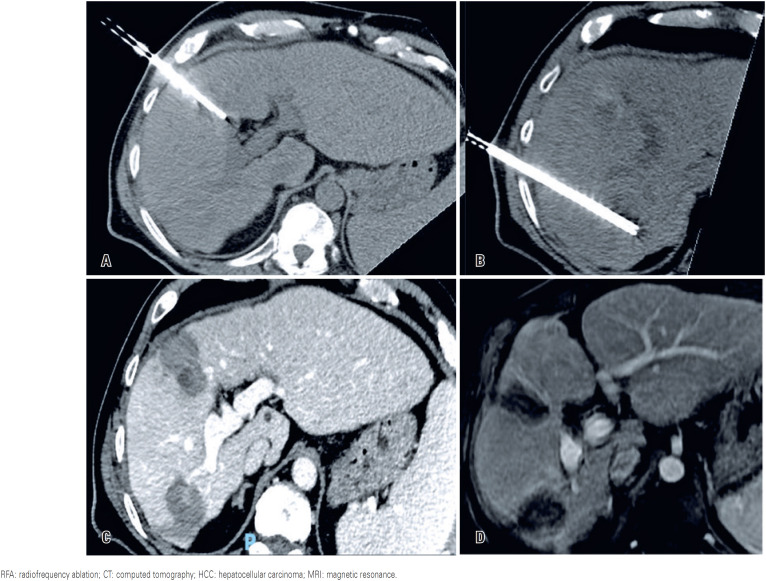
Axial CT-scan in a patient with early HCC treated with RFA. Intraoperative CT-scan showing the RFA needle positioning in a Liver Imaging Reporting And Data System 5 (LI-RADS- 5) at A) 2.3-cm lesion; B) 2.9-cm lesion; C) An axial CT-scan performed immediately after the procedure showing an optimal ablative zone and the absence of contrast enhancement in the tumors; D) An axial contrast-enhanced MRI performed 6 months after the procedure showing complete response

**Figure 2S:**
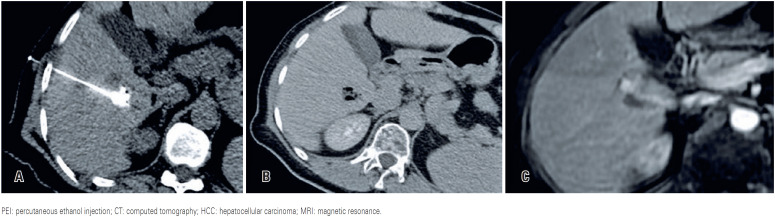
Axial CT-scan in a patient with early HCC treated with PEI. A) Intraoperative contrast injection and needle positioning in a Liver Imaging Reporting And Data System 5 lesion; B) An axial CT-scan performed immediately after the procedure; C) An axial contrast-enhanced magnetic resonance image performed 6 months after the procedure showing complete response

**Figure 3S:**
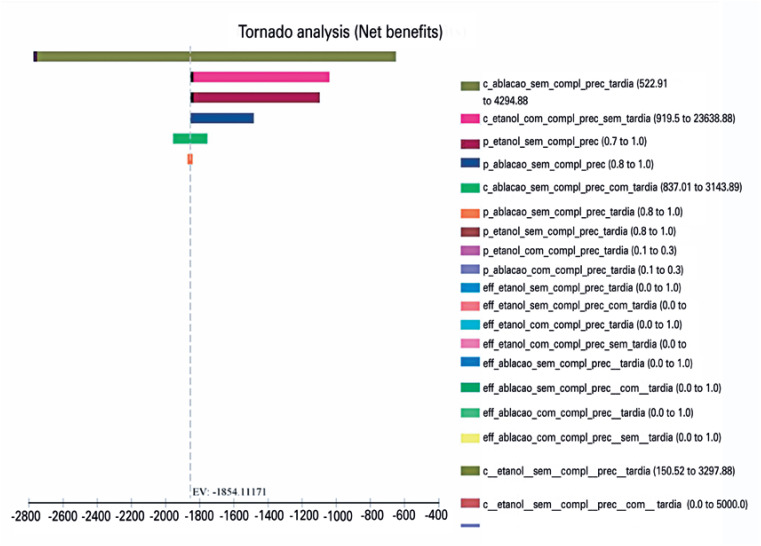
Tornado diagram for sensitivity analysis

## SUPPLEMENTARY MATERIAL

**Table 1S:** The complete objective response rates in each group

mRECIST objective response	RFA Group, n (%) (n=29)	PEI Group, n (%) (n=23)	Total, n (%) (n=52)	p value
Complete response	28 (96.5)	12 (52.2)	40 (77.0)	< 0.001
Partial response	1 (3.4)	5 (21.7)	6 (11.5)	
Stable disease	0 (0.0)	6 (26.1)	6 (11.5)	
Total nodules	29 (100.0)	23 (100.0)	52 (100.0)	

mRECIST: Modified Response Evaluation Criteria for Solid Tumors; PEI: percutaneous ethanol injection; RFA: radiofrequency ablation.

**Table 2S:** Summary quantitative and qualitative variables used in the tornado diagram for sensitivity analysis

Name	Description	Root Definition	Low Limit	High Limit
p_ablacao_sem_compl_prec	Probability radiofrequency ablation without early complications	1	0,8	1
p_etanol_sem_compl_prec	Probability percutaneous ethanol injection without early complications	0.8095	0,7	1
p_ablacao_sem_compl_prec_tardia	Probability radiofrequency ablation without early and late complications	0.9130	0,8	1
p_ablacao_com_compl_prec_tardia	Probability radiofrequency ablation with early and late complications	0	0,1	0,3
p_etanol_sem_compl_prec_tardia	Probability percutaneous ethanol injection without early and late complications	1	0,8	1
p_etanol_com_compl_prec_tardia	Probability percutaneous ethanol injection with early and late complications	0.2	0,1	0,3
eff_ablacao_sem_compl_prec_tardia	Effectiveness radiofrequency ablation without early and late complications	0.96	0	1
eff_ablacao_sem_compl_prec_com_tardia	Effectiveness radiofrequency ablation without early complications and with late complications	1	0	1
eff_ablacao_com_compl_prec_tardia	Effectiveness radiofrequency ablation with early and late complications	0	0	1
eff_ablacao_com_compl_prec_sem_tardia	Effectiveness radiofrequency ablation witht early complications and without late complications	0	0	1
eff_etanol_sem_compl_prec_tardia	Effectiveness percutaneous ethanol injection without early and late complications	.625	0	1
eff_etanol_com_compl_prec_tardia	Effectiveness percutaneous ethanol injection with early and late complications	1	0	1
eff_etanol_com_compl_prec_sem_tardia	Effectiveness percutaneous ethanol injection with early complications and without late complications	0.50	0	1
eff_etanol_sem_compl_prec_com_tardia	Effectiveness percutaneous ethanol injection without early complications and with late complications	0	0	1
c_ablacao_sem_compl_prec_tardia	Cost radiofrequency ablation without early and late complications	$ 1841.12	$ 522.91	$ 4294.88
c_ablacao_sem_compl_prec_com_tardia	Cost percutaneous ethanol injection without early complications and with late complications	$ 1990.45	$ 837.01	$ 3143.89
c_ablacao_com_compl_prec_sem_tardia	Cost percutaneous ethanol injection with early complications and without late complications	$ 0.00	$ 0.00	$ 5000.00
c_ablacao_com_compl_prec_tardia	Cost percutaneous ethanol injection with early and late complications	$ 0.00	$ 0.00	$ 5000.00
c_etanol_sem_compl_prec_tardia	Cost percutaneous ethanol injection without early and late complications	$ 1096.53	$ 150.52	$ 3297.88
c_etanol_sem_compl_prec_com_tardia	Cost percutaneous ethanol injection without early complications and with late complications	$ 0.00	$ 0.00	$ 5000.00
c_etanol_com_compl_prec_sem_tardia	Cost percutaneous ethanol injection with early complications and without late complications	$ 12279.19	$ 919.50	$ 23638.88
c_etanol_com_compl_prec_tardia	Cost percutaneous ethanol injection with early and late complications	$ 314.30	$ 200.00	$ 400.00
